# Ultrawideband electromagnetic metamaterial absorber utilizing coherent absorptions and surface plasmon polaritons based on double layer carbon metapatterns

**DOI:** 10.1038/s41598-021-02303-1

**Published:** 2021-11-29

**Authors:** Yongjune Kim, Pyoungwon Park, Jeongdai Jo, Joonsik Lee, Leekyo Jeong, Jonghwa Shin, Jeong-Hae Lee, Hak-Joo Lee

**Affiliations:** 1 Metamaterial Electronic Device Research Center, Hongik University, Seoul, 04066 South Korea; 2 Center for Advanced Meta-Materials, Daejeon, 34103 South Korea; 3 Department of Printed Electronics, Korea Institute of Machinery and Materials, Daejeon, 34103 South Korea; 4 Composite Research Division, Korea Institute of Materials Science, Changwon, 51508 South Korea; 5 Department of Materials Science and Engineering, Korea Advanced Institute of Science and Technology (KAIST), Daejeon, 34141 South Korea; 6 Department of Electronic and Electrical Engineering, Hongik University, Seoul, 04066 South Korea

**Keywords:** Electrical and electronic engineering, Microwave photonics, Polaritons

## Abstract

An ultrawideband electromagnetic metamaterial absorber is proposed that consists of double-layer metapatterns optimally designed by the genetic algorithm and printed using carbon paste. By setting the sheet resistance of the intermediate carbon metapattern to a half of that of the top one, it is possible to find an optimal intermediate metapattern that reflects and absorbs the EM wave simultaneously. By adding an absorption resonance via a constructive interference at the top metapattern induced by the reflection from the intermediate one, an ultrawideband absorption can be achieved without increasing the number of layers. Moreover, it is found that the metapatterns support the surface plasmon polaritons which can supply an additional absorption resonance as well as boost the absorption in a broad bandwidth. Based on the simulation, the $90\%$ absorption bandwidth is confirmed from 6.3 to 30.1 GHz of which the fractional bandwidth is 130.77$\%$ for the normal incidence. The accuracy is verified via measurements well matched with the simulations. The proposed metamaterial absorber could not only break though the conventional concept that the number of layers should be increased to extend the bandwidth but also provide a powerful solution to realize a low-profile, lightweight, and low cost electromagnetic absorber.

## Introduction

Electromagnetic (EM) absorbers are well-known components that can absorb the energy of the EM wave by converting it into heat based on the magnetic and dielectric losses^1,2^ or conduction loss^3–5^ of composite materials. They have been widely adopted in many fields such as the wireless communication to reduce the EM interferences^6,7^ or the stealth technology to minimize the radar cross section (RCS)^8,9^. Despite of excellent performances of the electrically conductive or magnetic composite materials, there exist some inherent constraints that the increments of the absorption bandwidths are limited^10,11^ or the weights of them are quite heavy to be applied for the mobile platforms. Also, relatively low durability may limit their application in maritime or atmospheric environments accompanying high temperature and/or humidity conditions.

To overcome these issues, metamaterials have attracted great attention, which are engineered structures that can realize extreme electromagnetic properties such as negative^12–14^ or zero constitutive parameters^15–17^, or peculiar wave controls such as electromagnetic cloaks^18–24^. Slightly different from the original viewpoint of metamaterials as isotropic^25,26^ or anisotropic^12–14^ effective media, the metamaterial absorbers can be modeled as critically coupled resonators^27,28^ of which the top and bottom sides of a dielectric substrate are covered by conductive patterns^29,30^, or one of the sides are blocked by a metallic layer^31–33^ or opened to the air^34,35^. Especially for the cases that utilize coupling of electric (*E*) fields to resonant currents on a lossy conductive pattern, the ohmic loss operates as a key factor for the absorption. The mechanism is also well known for the coherent perfect absorption utilizing two waves propagating toward opposite directions of each other^36–38^ or a single beam illuminated on a conductive film attached on the perfect magnetic conductor (PMC)^39^.

Even though the performances of the metamaterial absorbers have been verified successfully, there exists a limitation to extend the absorption bandwidth based on single-layer conductive patterns. It is partially due to the limited area on a plane to assemble multi-resonant structures at once. Even though the patterns combined with chip resistors can improve the bandwidth performance^40,41^, it may be not sufficient to cover the low and high frequency bands simultaneously. For instance, the fractional bandwidths satisfying − 10 dB reflectance are limited to 63.4% and 48.9% for the single-layer metamaterial absorbers utilizing double resonance resistive fan-shaped^40^ and eight-arms^41^ resonators, respectively. The fractional bandwidth is calculated by the ratio between the − 10 dB bandwidth and the center frequency of it. To support multiple resonances, multilayer structures can be alternatives, which consist of conductive patterns inserted among stacked dielectric substrates^42–45^. The concept opened the way to design broadband metamaterial absorber. However, there exist drawbacks that the total size of the structure becomes bulky and/or the fabrication process becomes complex as the number of the stacked layers is increased.

In this paper, an ultrawideband metamaterial absorber is proposed that consists of double-layer metapatterns printed using carbon paste. Based on the well-known configuration of the metamaterial absorber of which the bottom side is coated with a metallic layer, the main body consisted of two stacked acrylic plates on which the metapatterns composed with conductive square pixels are attached. By setting the sheet resistance of the intermediate metapattern less than that of the top one as well as optimizing the alignment of the pixels using the genetic algorithm^46–48^, the bandwidth satisfying the 90% absorption can be significantly enhanced without increasing the number of pattern layers above two. The optimized intermediate metapattern serves a multifunction not only absorbing the EM wave but also reflecting the EM wave backward. The reflected wave forms an additional constructive interference at the top metapattern, which can add an absorption resonance to the intrinsic resonances. In addition, it is confirmed that surface plasmon polaritons (SPPs) are supported, which can boost the broadband absorption. Based on the full-wave simulation, the bandwidth of 90% absorption was confirmed from 6.3 to 30.1 GHz of which the fractional bandwidth is 130.77% for the normal incidence. In addition, broad 80% absorption bandwidths were confirmed of which the fractional bandwidths are 137.41% and 130.94% with the incident angles up to $45^\circ$ and $60^\circ$ for the transverse electric (TE) and transverse magnetic (TM) polarizations, respectively. Based on the measurements well-matched with the simulation results in the X and Ku bands for the incident angles of $0^\circ$, $45^\circ$ and $60^\circ$, the performance of the proposed metamaterial absorber were verified. Based on the ultrawideband absorption covering from a part of C to a part of Ka band, the proposed metamaterial absorber could be applied to solve the electromagnetic interference problems of wireless and satellite communication and radar systems as well as to reduce RCS of surveillance and reconnaissance platforms.

## Design concept

**Figure 1:**
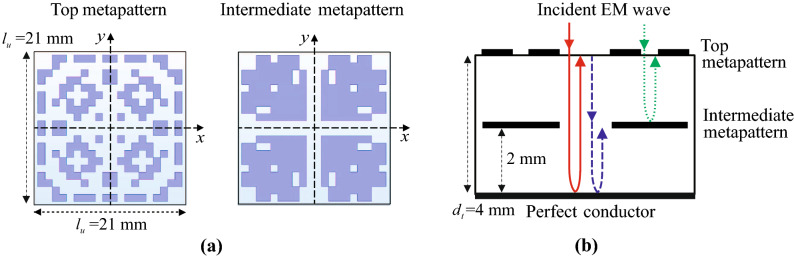
Schematic of metamaterial absorber composed with double-layer carbon metapatterns. (**a**) Optimized carbon metapatterns. Left-hand side: Top pattern, Right-hand side: Intermediate pattern. (**b**) Total structure and schematics of incident and reflected waves.

To design an ultrawideband absorber, a metamaterial structure is proposed as shown in Fig. 1. The structure consists of two carbon metapatterns shown in Fig. 1a, of which the design process will be discussed in the next section. One of the patterns, the intermediate one, is inserted between two stacked acrylic substrates, and the other is attached on the top of the structure as shown in Fig. 1b. The bottom of the total structure is blocked with the perfect conductor (PEC). The total thickness of the structure $d_t$ was chosen to be 4 mm for a direct comparison with previous low-profile X-band (8–12 GHz) metamaterial absorbers of which the thicknesses are about $0.2\lambda _g$ for the center frequency 10 GHz, where the wavelength in a substrate is indicated by $\lambda _g$^40,41^. The relative permittivity of the acrylic layer is about $2.6-i0.01$ at 10 GHz^49^, therefore, the thickness relative to the wavelength is $0.22\lambda _g$ that is comparable with the referenced one.

Similar with the concept of the coherent perfect absorber utilizing a constructive interference of the *E* field^36,37,39^, standing waves of which the *E* field intensities are maximized at the top or intermediate metapatterns can be utilized for the ultrawideband absorption. Figure 1b shows simplified schematics of the incident and reflected waves inside and outside the total structure. First, the EM wave incident on the top of the total structure interferes with the wave transmitting through the intermediate pattern after reflected backward from PEC. The interference is indicated by a red solid line in Fig. 1b. Because the wave suffers $\pi$ phase shift when it is reflected from PEC the maximum interference can be found when there exists a phase difference of $\pi$ between the incident and reflected waves. While the EM waves are reflected from the patterns or transmit through them, the phases of them can be changed abruptly by interacting with the metapatterns^50^. Therefore, the phase condition for the constructive interference can be achieved by designing the patterns optimally under the fixed configuration of the total structure. The design strategy will be discussed in the next section. Without consideration of the phase change owing the the metapatterns, the ideal design concept is described in following paragraphs.

Different from the Fabry–Pérot cavity of which the top and bottom are combined with highly reflective surfaces, the proposed top and intermediate metapatterns are highly resistive thin surfaces of which the thicknesses are lower than the skin depth of it at the working band. Therefore, incident EM wave partially transmit through as well as reflected by the metapatterns while it is perfectly reflected at the bottom of the total structure which is blocked by the perfect conductor (PEC). Hence, the constructive interferences can be formed at the height of the total structure $d_t$ under the condition $d_t= (2n-1)\lambda _n/4$, where *n* and $\lambda _n$ are a positive integer and wavelength in the metamaterial, respectively. The condition satisfies the phase difference of $(2n-1)\pi$ between the incident and reflected waves except the phase shift $\pi$ owing to the reflection. Based on it, the $n{\text {th}}$ harmonic frequency $f_n$ can be calculated as $f_n=(2n-1)c/4d_t$ using the relation $f_n=\lambda _n/c$, where *c* is the speed of light in the free space. Because the maximum interferences of the waves can be coupled to the surface electric currents induced on the metapattern, the power of EM waves can be converted to heat via ohmic losses. Because the electrically resistive carbon metapattern is analogous to an over damped harmonic oscillator which has a low quality (Q) factor^51^, the absorption by the metapatterns can guarantee a broadband absorption.

Second, the wave incident on and transmitting through the top metapattern interferes with the wave reflected from PEC at the location where the intermediate metapattern is inserted. The interference is indicated by a blue dashed line in Fig. 1b. Here, the intermediate pattern is located at the center of the total structure in the vertical direction. The location was determined under the assumption that the frequency of the constructive interference by the standing wave at the intermediate pattern is double of that of the interference at the top pattern. The resonance frequency of this mode $f'$ can be calculated as $f'=c/2d_t$, where *c* and $d_t$ are the speed of light in the free space and the height of the total structure, respectively. The second harmonic frequency of the constructive interference at the top metapattern is calculated as $f_2=3c/4d_t$ with $n=2$ that is triple of the fundamental one $f_1=c/4d_t$. Therefore, it can be expected that the frequency band of the constructive interference at the intermediate pattern may bridge the gap between the bands of the first and second harmonics of the constructive interferences at the top metapattern.

Third, the wave incident on the top metapattern interferes with the wave reflected from the intermediate metapattern. The interference is indicated by a green dot line in Fig. 1b. In this case, the wave reflected from the intermediate metapattern does not suffer the phase change of $\pi$ different from the wave reflected from PEC. Therefore, it is possible to extend the bandwidth utilizing the resonance frequency that does not overlap with those of the previous standing waves. Here, the sheet resistance of the intermediate pattern is set to 50 $\Omega$/sq that is a half of that of the top one 100 $\Omega$/sq. Based on the relation $\sigma =1/R_s\cdot d_p$, the conductivities $\sigma$ of the top and intermediate patterns can be calculated as 500 and 1000 S/m, respectively, where $d_p$ indicates the thickness of the  metapattern which is measured about 20 $\upmu$m. The sheet resistances of the top and intermediate metapatterns were determined by considering not only the moderate range of them for the absorbing performance^31^ but also the feasibility of fabrication determined by the sintering time and temperature of the carbon paste. By decreasing the resistivity of the pattern, that is inversely proportional to the conductivity, the absorption at the intermediate pattern may be slightly decreased while the reflection at the surface is increased. The trade-off relation may enable extending the absorption bandwidth by supplying an additional band while the absorption level in another band may be decreased. The mechanism provides a novelty that distinguishes the proposed metamaterial absorber from other one designed by the coupled-mode theory^52^. The later one considers multiple resonances in the similar way, however, the number of conductive patches have to be increased to be matched with the number of resonances. Moreover, the locations of them have to be optimized finely along the vertical direction which may increase the fabrication complexity.

## Design method and simulation results

**Figure 2:**
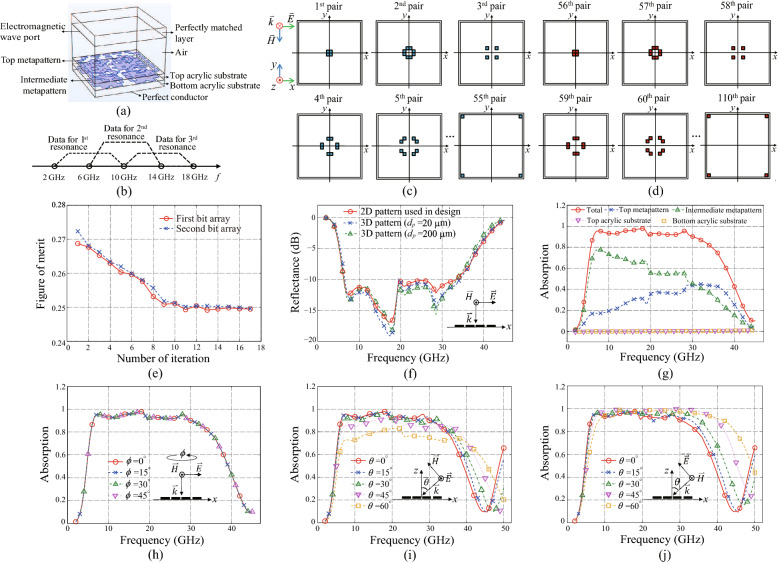
Schematics for designing optimal carbon metapatterns and simulation results of optimized metamaterial absorber. (**a**) Total structure and simulation setting. The side exterior boundaries of it are set to the periodic boundary condition. (**b**) Division of frequency domain into overlapping windows including three-points data. Combinations of pixel pairs for (**c**) top and (**d**) intermediate metapatterns. (**e**) Figure of merit vs number of iteration. (**f**) Simulated reflectances for normal incidence using 2D and 3D metapatterns. (**g**) Absorptions calculated at each part of the total structure and their total summation for normal incidence using 3D metapattern with $d_p$ = 200 $\upmu$m. (**h**) Absorptions calculated for horizontal rotations of TE polarization. Absorptions calculated for oblique incidences of (**i**) TE and (**j**) TM polarizations.

To design optimal metapatterns shown in Fig. 1a, the genetic algorithm^46–48^, i.e., a stochastic method that could find the ultimately optimized pattern mimicking the meiosis of the chromosome, was adopted. To this end, the top and intermediate layers were discretized into $20 \times 20$ square pixels of which the size is $1 \times 1$ $\text {mm}^2$. At the outer boundaries of the pixel arrays, a gap was added of which the width is 0.5 mm. The gap separates the unit cell of the absorber from the adjacent one as well as adds series capacitances between the unit cells which may assist inducing the resonant currents on the patterns. To design the metamaterial unit cell under the infinite-array condition, the periodic boundary condition was assigned to the side exterior boundaries of it. The simulation was conducted using the COMSOL Multiphysics, a commercialized finite element method (FEM) tool. The setting for the full-wave simulation is shown in Fig. 2a. The configuration and the material properties of the metamaterial absorber are the same with those introduced in the previous section. The top of the metamaterial absorber is filled with the air with a thickness of $\lambda _0/2$, where the $\lambda _0$ is the free space wavelength at 10 GHz, which is the center frequency of the target bandwidth which will be introduced below paragraph. At the top of the air box, the port irradiating the EM wave is located, and the perfectly matched layer is attached to truncate the simulation domain without any backward reflection.

As an initial step for the design, random bit arrays composed with one and zero were generated. To search the possible combination of bits sufficiently, the number of the randomly generated bit arrays was determined to be the same with the number of bits included in an array $N_{g}$^48^. Among them, a pair of bit arrays was selected as a dominant pair by competing the figure of merits (FOM) of them. To evaluate the absorbing performance in a broad bandwidth involving the low and high frequencies, FOM was calculated by averaging the reflectance of the metamaterial absorber in the frequency range from 2 to 18 GHz. The simulation method for the metamaterial absorber will be described in the next paragraph. For the efficient calculation of FOM, the reflectance was calculated at five frequency points with the interval of 4 GHz. The condition was determined heuristically by assuming that there exist three eigen frequencies in the band, that are matched to the three standing waves. Based on the study that estimates a resonance frequency with three-points data, it can be regarded that five-frequency points are the minimum for the efficient simulation by applying overlapping widows shown in Fig. 2b^53^. Here, it is supposed that the triple resonances will be found inside the targeted band if FOM caclculated with the five-frequency points are minimized. Even though the resonances are found outside the targeted band, they could influence on the absorption inside the windows owing to the low Q factors of the carbon metapatterns. Therefore, they can be found inside or near the target band where they can contribute to satisfying the criterion of FOM, i.e., − 10 dB, in the band.

For the full-wave simulation, each bit was matched to square pixels which are symmetrically located along the *x* and *y* axes as shown in Fig. 2c,d that show the pixel pairs located on the top and bottom acrylic layers, respectively. The pixels symmetrically located along the *x* and *y* axes were grouped as a pair. If the bit was one or zero, the matched pixels were filled with the carbon or air, respectively. This is the decoding process of a bit array. Because the total number of the pairs of the pixels $N_{g}$ included in both of the top and intermediate layers was 110, the same number of bits was included in the bit array. As described in the previous section, the sheet resistances of the carbon were set to 100 and 50 $\Omega$/sq for the top and intermediate metapatterns, respectively. The inset shows the polarization of the wave normally incident on the plane where the pixel arrays are located. To reduce the computational costs, the transition boundary condition was applied on the pixels, which can simplify the numerical analysis of the thin layer with the normal incidence condition^54^. By using it, a volumetric pixel can be replaced by a two-dimensional (2D) one including information of constitutive parameters of the material and thickness of it. Here, the relative permittivities of the carbon and air were set to 8.72, by referencing the properties of the carbon-black composite^55^, and 1, respectively. The maximum size of the mesh was set to $\lambda _{min}/5$, where $\lambda _{min}$ is the minimum wavelength of the EM wave in the band^56^.

After selecting the best pair of bit arrays by competing FOMs of the bit arrays in the initial set, the cross and mutation operations of the genetic algorithm were applied on them. To proceed the cross operation, a random point on the bit array was selected simultaneously for the pair. Then, the bits located after the selected point were exchanged with those included in the other bit array, and vice versa. In this paper, the cross operation was repeated 28 times by generating the same number of random points on the bit array, that is approximately a quarter of $N_{g}$. Next, the mutation was applied by selecting random points on each bit array. If the selected bit was one, it was converted to zero, and vice versa. Here, the mutations were applied five times for each new bit array generated by the cross operation. The full process of the genetic operations was repeated 110 times, that is the same number with $N_{g}$, using the selected best pair of bit arrays. Based on a new set of 110 bit arrays produced by the operations, FOMs of the metamaterial absorbers of which the patterns were matched with the bit arrays were calculated via the full-wave simulation. While calculating FOM, an optimal pair of bit arrays can be found by competing them with those of other bit arrays. After selecting the best pair of the metamaterial absorbers, the information of the patterns was encoded into bit arrays, and the same genetic operations were applied on them repeatedly. Then, a final pair of the bit arrays was selected by checking the convergence of FOM as shown in Fig. 2e. In this paper, the process was stopped at the seventeenth iteration. Between the two bit arrays included in the pair of the final step, an array was chosen of which the reflectance spectrum is wider than that of the other one. The selected array was matched to the metapatterns in Fig. 1a.

To verify the reliability of the numerical analysis based on the 2D structure with the transient boundary condition, the same structures were simulated using the three-dimensional (3D) metapatterns. Owing to the symmetry of the patterns along the *x* and *y* axes, the simulation results for the TE and TM polarizations have to be the same with each other for the normal incidence. Therefore, the reflectance was simulated using the TM polarization representatively as the inset of Fig. 2f. Here, the TE and TM polarizations are the cases that the *E* and magnetic (*H*) fields are perpendicular to the plane of incidence *x*o*z* plane, respectively, as described in Fig. 2c. First, the 3D patterns of which the heights are the same with those of the former 2D patterns were simulated with the same material properties. Even though there exists discrepancies between the results of 2D and 3D patterns near the null points of the spectrum in Fig. 2f, the levels are well matched near the − 10 dB reflectance. It means that the design utilizing the 2D patterns can provide pattern information satisfying the design criterion for the ultrawideband absorption. Moreover, to reduce the computational resources for the verification using the 3D patterns, e.g., the simulation time and the memory, another 3D patterns were simulated of which the heights were set to 200 $\upmu$m that is 10 times thicker than that of the previous one 20 $\upmu$m. By reducing the conductivities of the top and intermediate patterns to 50 and 100 S/m, respectively, that are 1/10 of those of the original pair, the resistances of the patterns can be maintained. In addition, frequencies where the skin depths^57^ of the carbons are equal to the thickness 200 $\upmu$m were calculated as 126.65 and 63.33 GHz for the conductivities of 50 and 100 S/m, respectively, by using

1 $$\begin{aligned} f=\frac{1}{\delta ^2 \pi \mu _0 \sigma }. \end{aligned}$$

The skin depth, permeability of free space, and conductivity are indicated as $\delta$, $\mu _0$, and $\sigma$, respectively. For the original pair of conductivities 500 and 1000 S/m of the 20 $\upmu$m patterns, the frequencies were calculated as 1.26 and 0.63 THz, respectively. From the calculations, it can be confirmed that the resistances of the scaled patterns do not change owing to the skin effect at the frequencies below 63.3 GHz. Therefore, the absorbing performance of the scaled patterns can be remained at the same levels with those of the original one at the frequency range. From Fig. 2f, it was verified that the tendency of the reflectance spectrum of the scaled patterns shows a good agreement with those of the other spectra. Even though there exist slight discrepancies near the null points, the spectra are well matched at the design criterion − 10 dB. Therefore, the full-wave simulation results in the following parts will be presented utilizing the patterns of which the thicknesses are 200 $\upmu$m.

**Figure 3:**
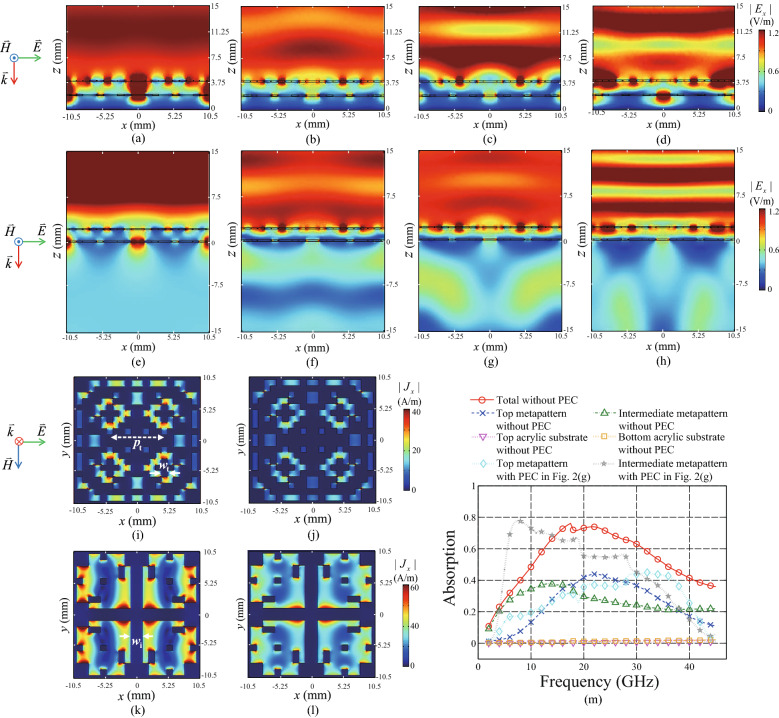
Magnitudes of electric field intensities along the x axis $|E_x|$ at $y=- 5.25$ mm with the perfect conductor (PEC) at the backside of the total structure for the normal incidence using 3D pattern with $d_p$ = 200 $\upmu$m at frequencies (**a**) 7.5 GHz, (**b**) 17.5 GHz, (**c**) 21 GHz, and (**d**) 28.5 GHz. $|E_x|$ at $y=-5.25$ mm with a half-infinite acrylic medium without PEC at frequencies (**e**) 7.5 GHz, (**f**) 17.5 GHz, (**g**) 21 GHz, and (**h**) 28.5 GHz. Magnitudes of surface current densities along the x axis $|J_x|$ on the top metapattern at frequency 7.5 GHz (**i**) With PEC and (j) Without PEC. $|J_x|$ on the intermediate metapattern at frequency 28.5 GHz. (**k**) With PEC and (**l**) Without PEC. (**m**) Absorptions calculated at each part of the total structure without PEC and their total summation. To compare the absorptions at the top and intermediate patterns before and after removing PEC, the results included in Fig. 2g are supplied.

From Fig. 2f, the bandwidth of − 10 dB reflectance can be confirmed from 6.3 to 33.2 GHz of which the fractional bandwidth is 136.2% for the normal incidence. The fractional bandwidth is calculated by the ratio between the − 10 dB bandwidth and the center frequency of it. Here, it is found that the bandwidth is extended beyond the targeted band that is limited to 18 GHz. This is because there is a limitation to locate the triple resonance frequencies in the targeted band at once. While two resonance frequencies are found at 7.5 and 17.5 GHz inside the targeted band, the highest resonance frequency is shown at 28.5 GHz in Fig. 2f. Even though the highest frequency is located at the outside of 18 GHz, it can contribute to reducing FOM in the targeted band owing to the nature of the low Q of the carbon metapatterns. Another resonances appeared at 17.5 and 21 GHz will be discussed in this section using Fig. 3. From the results, it can be confirmed that the ultrawideband absorption can be achieved not only by the proposed configuration of the metamaterial absorber but also via the deep search for the optimal patterns using the genetic algorithm. It is possible to extend the bandwidth by increasing the number of widows at the high frequency region. However, it may degrade the absorption performance at the low frequency region because the resonance frequencies can be shifted upward.

**Figure 4:**
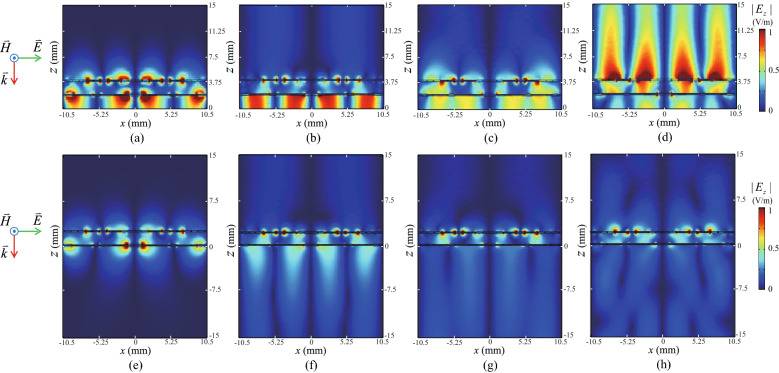
Magnitudes of electric field intensities along the z axis $|E_z|$ at $y=-\,5.25$ mm with the perfect conductor (PEC) at the backside of the total structure at frequencies (**a**) 7.5 GHz, (**b**) 17.5 GHz, (**c**) 21 GHz, and (**d**) 28.5 GHz. $|E_z|$ at $y=-\,5.25$ mm with a half-infinite acrylic medium without PEC at frequencies, (**e**) 7.5 GHz, (**f**) 17.5 GHz, (**g**) 21 GHz, and (**h**) 28.5 GHz.

Because the reduction of the specular reflection may not be coincided with the absorption of the incident power owing to diffusions by the metapatterns^45,58^, the absorption bandwidth was calculated precisely. To this end, the dissipated powers by the ohmic and dielectric losses were calculated by integrating the dissipated power densities with respect to volume of the carbon patterns and acrylic substrates via the full-wave simulation results^51,54^. Because the calculation of the dissipated power is not available using the approximated 2D patterns of which the volume integral cannot be calculated, the specular reflectance has to be utilized for the design process. On the other hand, it is available calculating volume integral using the 3D patterns with the thickness of 200 $\upmu$m. After calculating the total summation of the dissipated power inside the 3D patterns and acrylic substrates, the absorption was evaluated by dividing the total dissipated power by the input power. The input power $P_{in}$ can be calculated by integrating the input time-averaged power density over the area of the unit cell,

2 $$\begin{aligned} P_{in}=\frac{1}{2\eta _0} E_0^2 l_u^2 \text {cos}\theta , \end{aligned}$$

where the electric field intensity, the length of the unit cell, the vertical incident angle, and the intrinsic impedance of the air are indicated by $E_0$, $l_u$, and $\theta$, $\eta _0$, respectively. Here, $E_0$, $l_u$, $\theta$, and $\eta _0$ were set to 1 V/m, 21 mm, $0^\circ$, and 377 $\Omega$, respectively. Figure 2g shows the calculated absorptions for each part of the metamaterial absorber as well as the total summation of them. From the calculated absorption for each part, it can be found that the input power is absorbed dominantly by the patterns via the ohmic losses while the dielectric losses inside the acrylic substrates are minor. The absorption spectra of the top and intermediate metapatterns will be discussed in this section by matching them with the *E* field intensities inside and outside the metamaterial absorber and surface current densities on the metapatterns which will be shown in Figs. 3 and 4. Based on the total absorption, the bandwidth of $90\%$ absorption can be confirmed from 6.3 to 30.1 GHz of which the fractional bandwidth is 130.77% for the normal incidence. From the fractional bandwidth as broad as that of the − 10 dB reflectance, it can be validated that the reduction of the reflected wave is mainly obtained by the absorption of the metamaterial absorber.

**Table 1:** Comparisons of simulation results, thicknesses, and fabrication complexities of multilayer electromagnetic absorbers.

	− 10 dB reflectance bandwidth		$d_t/\lambda _l$	$d_t$ (mm)	Number of pattern layers	Fabrication complexity
	GHz	$\%$				
^42^	13.3 (4.96–18.22)	114.4	0.076	4.6	2	Moderate
^43^	30.4 (7–37.4)	137	0.089	3.8	3	Moderate
^44^	6.3 (1.5–7.8)	135.5	0.15	30.15	3	High
^45^	35.5 (7.5–43)	140.6	0.082	3.27	2	High
This work	26.9 (6.3–33.2)	136.2	0.084	4	2	Low

By comparing the performance with those of other multilayer metamaterial absorbers as Table 1, the excellence of the proposed metamaterial absorber can be verified. Here, the − 10 dB reflectance bandwidths are compared because the absorptions reported in other papers were evaluated by subtracting the linear-scale reflectance, i.e., $|\Gamma |^2$ or $|\text {S11}|^2$, from unity, where the reflection coefficient is indicated by $\Gamma$ or S11. From Table 1, it can be confirmed that the fractional bandwidth of the proposed one is comparable with a record-high bandwidth reported thus far^45^. In addition, it can be found that not only the total thickness $d_t$ but also the ratio between $d_t$ and the wavelength of the lowest frequency of the band $\lambda _l$ are as low as those of other high-level multilayer metamaterial absorbers. Most importantly, there is a strong advantage on the proposed metamaterial absorber that the fabrication process is relatively simple compared with other multilayer structures. The metamaterial absorber composed with double-layer metapatterns utilizing chip resistors may require burdensome soldering process^42^, and the absorber composed with triple resistive layers may need an additional effort to fabricate and assemble the three layers that have different thicknesses each other^43^. The triple-layer graphene oxide and double-layer graphene metamaterial absorbers may need time-consuming and/or complex fabrication method such as reduction procedure for the graphene oxide^44^ and annealing and reduction procedures for patterning the graphene^45^, respectively. The fabrication process of the proposed metamaterial absorber will be described in the next section.

Furthermore, it is verified that the absorption is not changed when the polarization of the incident wave rotates horizontally along the *z* axis. From Fig. 2h, completely overlapped absorption spectra can be found for the polarizations rotated from the TM polarization with an interval of $15^\circ$ up to $\phi =45^\circ$. Owing to the symmetry, the angle of rotation is limited to $\phi =45^\circ$. It can be regarded that the symmetry guarantees the invariance of superposition of responses of *E* fields decomposed in to *x* and *y* axes. In addition, full-wave simulations were performed for various vertical incident angles with two orthogonal TE and TM polarizations to investigate the absorbing performance for the oblique incidences. The vertical incident angle $\theta$ in Eq. (1) was swept from $0^\circ$ to $60^\circ$ with an interval of $15^\circ$. Figure 2i,j show the simulation results of the oblique incidences for the TE and TM polarizations, respectively. The insets included in Fig. 2i,j show the schematics of the oblique incidences utilizing the TE and TM polarizations, respectively. As shown in Fig. 2i,j, the absorbing performances for the two orthogonal polarizations have to be varied for the oblique-incidence scenarios. It is because the patterns of the metamaterial absorber were optimized under the condition of the normal incidence. The wave of the normal incidence does not have field components along the *z* axis while the oblique incidences of the TE and TM polarizations have the *H* and *E* fields along the *z* axis, respectively.

By considering the performance degradation of the metamaterial absorber for the oblique incidences, the reflectance bandwidths were evaluated with an alleviated criterion of 80% absorption. The bandwidths of 80% absorptions were confirmed from 6.4 to 34.5 GHz of which the fractional bandwidth is 137.41% with the incident angle up to $45^\circ$ for the TE polarization as shown in Fig. 2i. In addition, the bandwidths were confirmed from 7.2 to 34.5 GHz of which the fractional bandwidth is 130.94% with the incident angle up to $60^\circ$ for the TM polarization as shown in Fig. 2j. By comparing both of the results, it can be revealed that the absorption performance for the TM polarization is better than that for the TE one from the perspective of the range of incident angle. Because SPP can be induced for the TM polarization and boost the absorption^59,60^, the range of incident angle can be extended for the TM polarization of which the *H* field is fixed to be parallel with the surface of the metapattern. Simulation results supporting the description will be shown at the last paragraph of this section.

To clarify the absorbing mechanism described in the previous section, the field and current distributions inside and outside of the metamaterial absorber are plotted in Fig. 3. To verify the three constructive interferences at the top and intermediate metapatterns, the magnitudes of *E* fields along the *x* axis ($|E_x|$) are plotted in Fig. 3a–d on the *x*o*z* plane at $y=-l_u/4$, where $l_u$ is 21 mm. The incident polarization is the same with the inset of Fig. 3a. The field distributions for the total structure blocked by PEC were plotted in Fig. 3a–d at the frequencies of 7.5, 17.5, 21, and 28.5 GHz, respectively, where the nulls are located in the reflectance spectrum of Fig. 2f. Figure 3e–h show the field distributions when the bottom of the structure is combined with a half-infinite acrylic medium after removing PEC. To this end, a perfectly matched layer utilizing the properties of the acrylic layer was assigned at the end of the acrylic space $z=-15$ mm, that can truncate the simulation domain without any reflection. By comparing Fig. 3a–d with Fig. 3e–h, respectively, the effect of PEC can be distinguished clearly.

From Fig. 3a, the constructive interferences at the top metapattern are found at (*x*, *z*) = (0 mm, 4 mm), (± 4.5 mm, 4 mm), (± 7 mm, 4 mm), and (± 10 mm, 4 mm) for the frequency 7.5 GHz. By comparing $|E_x|$ of Fig. 3a with that of Fig. 3e at each location described above, it can be clearly considered that the interferences are related with the reflection by PEC. Even though the locations (*x*, *z*) = (± 4.5 mm, 2 mm) and (± 7 mm, 2 mm) are blocked by the intermediate metapattern, the EM wave of which the frequency is 7.5 GHz can transmit through it. This is because the skin depth $\delta$ of the intermediate metapattern is 581.2 $\upmu$m at 7.5 GHz while the thickness of the pattern is 200 $\upmu$m. The skin depth is calculated as $\delta =\sqrt{1/\pi f \mu _0 \sigma }$, where the frequency, permeability of free space, and conductivity are indicated as *f*, $\mu _0$, and $\sigma$, respectively^57^. To support the description that the absorption resonance at 7.5 GHz is owing to the interference related with the reflection by PEC, the magnitudes of the surface electric current densities along the *x* axis ($|J_x|$) induced on the top metapattern are compared before and after removing PEC as shown in Fig. 3i,j, respectively. Because the ohmic losses are proportional with the magnitudes of the current densities, $|J_x|$ of Fig. 3i larger than that of Fig. 3j supports the absorbing mechanism. The result is also matched with the first absorption peak at 7.5 GHz of the graph indicated by top metapattern in Fig. 2g. The large absorption peak at the same frequency of the graph indicated by intermediate metapattern will be discussed at the end of this section using Fig. 4. Because resonances at (*x*, *z*) = (0 mm, 2 mm) and (*x*, *z*) = (± 10 mm, 2 mm) in Fig. 3a can be found at the same locations in Fig. 3e without PEC, they can be regarded as additional resonances by the direct coupling between the EM wave and the carbon pattern^52^.

From Fig. 3d, the constructive interferences at the intermediate metapattern are found at (*x*, *z*) = (0 mm, 2 mm) and (± 10 mm, 2 mm) for the frequency 28.5 GHz. By comparing $|E_x|$ of Fig. 3d with that of Fig. 3h at each location described above, it can be clearly considered that the interferences are related with the reflection by PEC. Besides, the magnitudes of the surface currents $|J_x|$ induced on the intermediate metapattern are compared before and after removing PEC as shown in Fig. 3k,l, respectively. From the enhanced $|J_x|$ of Fig. 3k compared with that of Fig. 3l, the absorption mechanism can be clearly verified. The result is also matched with the third absorption peak at 28.5 GHz of the graph indicated by intermediate metapattern in Fig. 2g. The large absorption at the same frequency confirmed at the graph indicated by top metapattern will be discussed at the end of this section using Fig. 4. The interference is established at the frequency 28.5 GHz much higher than the double of the lowest resonance frequency 7.5 GHz. It is not in accord with the assumption in the previous section owing to the abrupt phase modulations by the interaction between the EM wave and the metapatterns. Nonetheless, the gap between the two frequencies is bridged by the other absorption resonance which will be described in the next paragraph.

The other constructive interferences at the top metapattern are found at (*x*, *z*) = (± 4.5 mm, 4 mm) and (*x*, *z*) = (± 7 mm, 4 mm) in Fig. 3c,g with and without PEC, respectively, for the frequency 21 GHz. From the similar responses in Fig. 3c,g at the locations described above, it can be considered that the responses are not affected critically by PEC, and originated from the reflection by the intermediate metapattern. To clarify the mechanism, the absorption at each part of the total structure is calculated after removing PEC. From the absorption calculated at the top pattern as well as the total summation of the absorptions in Fig. 3m, a resonance peak can be confirmed at 22 GHz, which are almost matched with the third null of reflectance in Fig. 2f and the third absorption crest of the graphs indicated by top metapattern in Fig. 2g at 21 GHz. The slight frequency shift is caused by the changes of the absorption spectra of the metappaterns after combining PEC at the bottom. The degradation of the level of the graph indicated by top metapattern in Fig. 2g near the resonance frequency is owing to the increased impedance mismatch after combining PEC at the bottom. It can be confirmed by comparing the magnitude of the standing wave of Fig. 3c with that of Fig. 3g at the outside of the metamaterial absorber filled with the air. Here, the second harmonic with $n=2$ of the first resonance shown at 7.5 GHz may be supported near the frequency 22.5 GHz following the condition $f_n=(2n-1)c/4d_t$ discussed in the previous section, where *n*, *c*, and $d_t$ are a positive integer, the speed of light in the free space, and the height of the total structure, respectively. However, it cannot be distinguished clearly because the frequency is overlapped with the constructive interference related with the reflection from the intermediate metapattern shown at 22 GHz. Besides, it can be confirmed from Fig. 3m that the absorption by the intermediate metapattern at 17.5 GHz is larger than that at 21 GHz regardless of existence of PEC even though $|E_x|$ distributions inside the metamaterial absorber of Fig. 3b,f are similar with those of Fig. 3c,g, respectively. The mechanism that causes the difference will be discussed in the next paragraph using Fig. 4.

To explain the absorptions at the top and intermediate patterns shown in Fig. 2g and 3m completely, the magnitudes of *E* fields along the *z* axis $|E_z|$ are plotted in Fig. 4a–d on the same plane of Fig. 3a–d. From Fig. 4a–d, strongly resonant electric fields polarized along the *z* axis can be found at all of the resonant frequencies 7.5, 17.5, 21, and 28.5 GHz found in Fig. 2f. These additional resonances are SPPs which can be induced on the slits parallel with *H* field such as gaps located at (*x*, *y*) = (± 4.5 mm, ± 3.5 mm) and (± 4.5 mm, ± 5.5 mm) on the top metapattern in Fig. 3i  as well as gap located at *x* = 0 mm on the intermediate metapattern in Fig. 3k^61^. Because the fields are coupled to the surface current densities, it can be considered that SPPs are one of the key factors that enhance the absorbing performance of the proposed metamaterial absorber. Figure 4e–h show distributions of $|E_z|$ when the bottom of the structure is combined with the half-infinite acrylic medium after removing PEC by utilizing the same simulation setting of Fig. 3e–h. By comparing Fig. 4a–d with Fig. 4e–h, respectively, it is clearly found that SPPs can be boosted under the configuration of the metamaterial absorber accompanying PEC at the bottom of the total structure. This is owing to additional SPPs induced by the reflected EM waves from PEC^61^. The frequencies of SPPs are shifted upward when the geometric parameters such as the width of slit and the distance between slits are reduced^61^. The width of slit included in the intermediate metapattern indicated by $w_i$ in Fig. 3k is wider than that included in the top metapattern indicated by $w_t$ in Fig. 3i. In addition, the distance between the slits of the intermediate one is the same with the length of the unit cell while that of the top one indicated by $p_t$ in Fig. 3i is smaller than the length of the unit cell. Therefore, the resonance frequencies of SPPs induced on the intermediate metapattern are found at relatively low frequencies 7.5 and 17.5 GHz as shown in Fig. 4a,b, respectively. On the other hand, the SPP resonance on the top pattern is confirmed at relatively high frequency 28.5 GHz shown in Fig. 4d. Besides, SPP shown in Fig. 4b stronger than that of Fig. 4c supports the result that absorption via the intermediate metapattern at 17.5 GHz is higher than that at 21 GHz. From the result, it can be considered that the resonance at 17.5 GHz shown in Fig. 2f,g is supplied by SPP at the intermediate metapattern.

## Fabrication and measurements

For the experimental verification, the metapatterns were printed on the polyimide (PI) film using carbon paste (Changsung Nanotech, ECOSIL-CP101D) via the well known silk-screen method^62^. The carbon paste consists of 25–30 wt% carbon powder and 70–75 wt% solvent composed with a synthetic resin, ethyl and buthyl carbitols, and additives. The thickness of the PI film is 75 $\upmu$m. To fabricate the top and intermediate patterns on the PI film with the sheet resistances 100 and 50 $\Omega$/sq, respectively, the printed patterns were sintered on the hot plate during 10 minutes under the temperatures 90 $^\circ$C and 180 $^\circ$C, respectively. The measurement of the sheet resistances of the printed patterns will be discussed in the “Methods” section.

**Figure 5:**
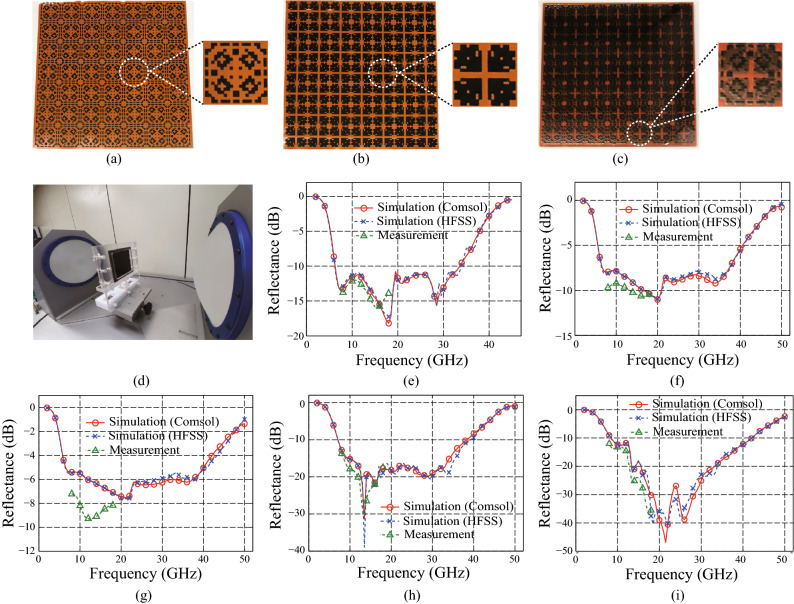
Fabricated metamaterial absorber, measured reflectances, and simulated results via Comsol multiphysics and HFSS using 3D metapatterns with $d_p$ = 200 $\upmu$m. Fabricated (**a**) top and (**b**) intermediate carbon metapatterns, and (**c**) total structure of the proposed metamaterial absorber. (**d**) Measurement system. (**e**) Measured and simulated reflectances for the normal incidence. Measured and simulated reflectances for TE polarization with incident angles (**f**) $\theta =45^\circ$ and (**g**) $\theta =60^\circ$. Measured and simulated reflectances for TM polarization with incident angles (**h**) $\theta =45^\circ$ and (**i**) $\theta =60^\circ$.

The printed top and intermediate carbon metapatterns are shown in Fig. 5a,b, respectively. Each printed pattern was attached on the 2 mm-thick acrylic layer. Then, the acrylic layer with the top pattern was stacked on the intermediate pattern attached on the other layer. The bottom side of the total structure was blocked by a copper sheet of which the thickness is 35 $\upmu$m. Because the skin depth of the copper is calculated as 0.833 $\upmu$m at the lowest frequency of the − 10 dB reflectance band $f=6.3$ GHz, the thickness 35 $\upmu$m of the copper sheet used for the experimental verification is sufficient for the perfect reflection. To attach the layers, an adhesive polyethylene terephthalate (PET) film was used of which the thickness is 95 $\upmu$m including the adhesive layer. The fabricated total structure is shown in Fig. 5c. To measure the reflectance of the fabricated metamaterial absorber, a measurement system shown in Fig. 5d was used. Because the system is composed with a pair of lens-horn antennas that have limited bandwidths, the measurement cannot be done for the entire bandwidth. In this paper, the reflectance was measured from 8 to 18 GHz using the lens-horn antennas customized for X and Ku bands.

Figure 5e shows a measured reflectance at the specular direction for the normal incidence. Because it was limited to measure the reflectance for the whole range of reflection angle via the measurement system, the metamaterial absorber was verified by comparing the simulated and measured reflectances at the specular direction. To measure the reflectance under the same condition with the simulation shown in Fig. 2f, the TM polarization was used. As shown in Fig. 5e, the measurement is well matched with the simulation using the 3D metapatterns of which the thicknesses are 200 $\upmu$m in the frequency range from 8 to 18 GHz. A slight down shift found at the measurement may be owing to increment of the thickness of the total structure by using the PI and adhesive PET films. To verify the accuracies at the other frequency ranges that cannot be measured via the system, additional full-wave simulation results were supplied by using another commercialized FEM based software HFSS. From the well-matched full-wave simulation results in the ranges from 2 to 8 GHz as well as from 18 to 45 GHz, the performance of the metamaterial absorber can be verified clearly in the entire frequency range.

To verify the absorbing performances for the oblique incidences, the measurements were conducted for the various incident angles with the two orthogonal polarizations. Due to the volumetric feature of the lens-horn antennas, the smallest incident angle was determined to $45^\circ$ that allows the nearest arrangement between two antennas with a rotational interval of $15^\circ$. The measurements for the TE and TM polarizations with the incident angles of $45^\circ$ and $60^\circ$ are shown in Fig. 5f,g and Fig. 5h,i, respectively. From the measurements well matched with the simulations for both of the two polarizations, the electromagnetic absorbing performance for the oblique incidences can be verified. Even though there exists a difference between the simulation and measurement especially for the incident angle $60^\circ$ with the TE polarization owing to fabrication and/or measurement inaccuracies such as errors on printed patterns and sheet resistances and/or an alignment of the antennas, respectively, the absorbing performance of the proposed metamaterial absorber can be verified by the well matched trends between simulations and measurements. In addition, the absorbing performances for the various incident angle could be validated via the well matched full-wave simulation results calculated by two different commercialized tools not only in the frequency range from 8 to 18 GHz but also from 2 to 8 GHz as well as from 18 to 50 GHz that were not measured by the system.

## Conclusion

An ultrawideband double-layer metamaterial absorber composed with two carbon metapatterns is proposed that can utilize quadruple absorption resonances. The resonances can be induced by the strong coupling between the constructive interferences of the *E* fields and the carbon metapatterns as well as SPPs boosted by the reflected EM wave from PEC. To achieve quadruple resonances without increasing the number of pattern layers above two, an additional constructive interference at the top metapattern is supplied to the intrinsic resonance at each metapattern by increasing the reflectance from the intermediate metapattern. In addition, an absorption peak is added utilizing a SPP resonance at the intermediate metapattern. To achieve the optimal carbon metapatterns, the genetic algorithm is applied efficiently by adopting a concept of overlapping windows composed with three-points data. As a result, the ultrawideband absorption was achieved not only by assembling the quadruple resonances inside and outside of the target-frequency band but also by utilizing absorption via the broadband SPP. Based on the proposed mechanism, the bandwidth that satisfies 90% absorption can be achieved from 6.3 to 30.1 GHz of which the fractional bandwidth is 130.77% for the normal incidence. Also, broad 80% absorption bandwidths were confirmed of which the fractional bandwidths are 137.41% and 130.94% with the incident angles up to $45^\circ$ and $60^\circ$ for the TE and TM polarizations, respectively. The accuracy of the proposed metamaterial absorber was verified from the measurements well matched with the simulation results as well as comparisons of two different types of full-wave simulation results. Most importantly, the screen printing method utilized in this paper guarantees the lowest complexity of the fabrication process among methodologies adopted to fabricate multilayer structures thus far. Because the acrylic substrate is transparent, the ultrawideband transparent metamaterial absorber could be realized by fabricating the metapatterns and perfect reflector based on transparent conductive materials such as graphene and indium tin oxide (ITO), respectively. This remains as a potential future study. In addition, the proposed design method may break through the conventional concept that the number of absorption resonances coincides with that of layers. Finally, the proposed metamaterial absorber may provide a powerful solution not only for broadening the bandwidth and the range of incident angle but also for reducing the number of layers, total thickness, weight, and cost of the EM absorber.

## Methods

To measure the sheet resistances of the printed carbon metapatterns, square carbon patches were used that have sufficiently large area. Here, $4 \times 4$ $\text {cm}^2$ patches were used. Even though the metapatterns and the patches were fabricated simultaneously under the same conditions, the thicknesses of the patches may be slightly thinner than those of the metapatterns owing to the spread of the carbon paste during the sintering process. It can make the sheet resistances of the patches higher than the target values. Therefore, the sintering temperatures 90 $^\circ$C and 180 $^\circ$C were determined to make the sheet resistances of the patches 5–15% higher than the target values. Using the four-point probe method^35,63^, the average sheet resistances of the printed carbon patches and their standard deviations were measured as 111.65 and 6.59 Ω/sq for the top metapattern, and 52.63 and 1.94  $\Omega$/sq for the intermediate metapatterns, respectively.
